# Paper-Based Multiplexed Colorimetric Device for the Simultaneous Detection of Salivary Biomarkers

**DOI:** 10.3390/bios11110443

**Published:** 2021-11-10

**Authors:** Tania Pomili, Paolo Donati, Pier Paolo Pompa

**Affiliations:** 1 Nanobiointeractions & Nanodiagnostics, Istituto Italiano di Tecnologia (IIT), Via Morego 30, 16163 Genova, Italy; tania.pomili@iit.it (T.P.); paolo.donati@iit.it (P.D.); 2 Department of Chemistry and Industrial Chemistry, University of Genova, Via Dodecaneso 31, 16146 Genova, Italy

**Keywords:** point-of-care, paper-based, multiplexing, colorimetric test, saliva

## Abstract

In this study, we describe a monolithic and fully integrated paper-based device for the simultaneous detection of three prognostic biomarkers in saliva. The pattern of the proposed multiplexed device is designed with a central sample deposition zone and three identical arms, each containing a pre-treatment and test zone. Its one-step fabrication is realized by CO_2_ laser cutting, providing remarkable parallelization and rapidity (ca. 5 s/device). The colorimetric detection is based on the sensitive and selective target-induced reshaping of plasmonic multibranched gold nanoparticles, which exhibit a clear spectral shift (and blue-to-pink color change) in case of non-physiological concentrations of the three salivary biomarkers. A rapid and multiplexed naked-eye or smartphone-based readout of the colorimetric response is achieved within 10 min. A prototype kit for POCT testing is also reported, providing robustness and easy handling of the device.

## 1. Introduction

The development of point-of-care tests (POCTs) has attracted great interest in the healthcare system, due to their rapidity (near-patient tests with real-time results), ease-of-use, and versatility in terms of target detection. POCTs can be fabricated by a wide range of low-cost materials, including paper, which has been demonstrated to be effective in several recent studies, due to its numerous advantageous features [1,2,3,4,5,6]. Paper-based POCTs may be easily folded to form suitable microchannels surrounded by hydrophobic walls, allowing for a controlled flow of the biological/analytical fluid under test. The hydrophilic pathway and hydrophobic walls can be obtained through a variety of methods, such as inkjet printing, wax printing, laser treatment, and cutting [7,8,9,10]. These approaches empower paper with several in-situ operations and functionalities, such as sample filtration, separation, multistep reactions, and colorimetric detection of target analytes. Most of the currently available paper-based POCTs, however, allow the evaluation of single targets, such as the paper-based test strips for pregnancy or the COVID-19 rapid antigen tests. Multiplexed analyses, on the other hand, are subject to increasing interest, as they offer superior clinical information, while saving on reagents, time, and costs [11,12,13,14]. Multiplexed assays, in fact, provide the ability to identify, in one single analysis, different parallel biomarkers or specific combinations of them, improving the overall clinical value of the test [15,16]. Moreover, their possible integration with nanomaterials may further increase their diagnostic potential, due to the possibility to boost device performance [17], especially in terms of sensitivity and long-term stability at ambient conditions. In this regard, it is worth mentioning that nanomaterials offer remarkable optical detection schemes due to their size-, shape-, and aggregation-dependent plasmonic properties (e.g., in gold nanoparticles) [18,19,20,21,22], alongside their innovative nanozyme functionalities that can provide both the replacement of biological enzymes, with higher stability and efficiency at ambient conditions, and sensitivity enhancement due to signal amplification [23,24,25,26,27,28]. An increased nanoplasmonics/nanocatalysis sensitivity presents greater opportunities for frequent near-patient screenings, enabling the use of non-invasive biological fluids (such as saliva, urine, or sweat), in which biomarker concentration is typically 1–2 orders of magnitude lower than in the blood [29,30,31,32].

In this work, we report on a monolithic paper-based device for the simultaneous colorimetric detection of three model salivary biomarkers. The assay is performed on real saliva samples by depositing a single drop in the central zone of the device and exploiting controlled reactions in the microfluidic paper channels, followed by the target-induced reshaping of multibranched gold nanoparticles. The proposed device allows for a rapid naked-eye or smartphone-based readout of the colorimetric response, representing a promising candidate biosensor for multiplexed analyses of prognostic biomarkers in non-invasive fluids.

## 2. Materials and Methods

### 2.1. Chemicals and Materials

Sodium iodide (NaI, for analysis EMSURE^®^ ISO, Reag. 1.06523, Merck-Sigma-Aldrich), D-(+)-Glucose (C6H12O6 ACS reagent, G5767), Glucose Oxidase from *Aspergillus* (Type X-S, lyophilized powder, G7141), Sodium L-Lactate (United States Pharmacopeia (USP) Reference Standard, 1614308), Lactate Oxidase from *Aerococcus viridans* (lyophilized powder, L9795), Cholesterol (Sigma Grade, ≥99%, C8667), Triton™ X-100 (laboratory grade, X100), Cholesterol Oxidase from microorganisms (lyophilized powder, ≥50 units/mg protein, recombinant expressed in *E. coli*, C8868), Sucrose (BioUltra, for molecular biology, ≥99.5% (HPLC), 84097), D-(+)-Maltose monohydrate (powder, BioReagent, ≥98%, M5895), D-(−)-Fructose (≥99%, F0127), D-Lactose monohydrate (BioUltra, ≥99.5% (HPLC), 61339), Sodium phosphate monobasic (BioReagent, for molecular biology, anhydrous, ≥98%, S3139), Sodium phosphate dibasic (ACS reagent, ≥99.0%, S9763) and Whatman^®^ qualitative filter paper (Grade 1, diam. 110 mm, WHA1001110) were purchased from Merck-Sigma-Aldrich. MPI™ 3000 Gloss High Opacity Series (95 µM thickness) was purchased from Avery Dennison^®^. Whatmann Fusion 5 (roll, 8151-9915) was purchased from Cytiva. 96W PE Sealing film (adhesive, optical, Corning^®^, 96W, 734-1756 was purchased from VWR. Lasercut61 was used for drawing microfluidic channels on the paper.

### 2.2. Design and Fabrication of the Analytical Device

For the fabrication of the multiplexed device, AutoCAD software was employed to design the fluidic pattern of the paper. Subsequently, the template was transferred to a sheet of Whatman No.1 Chromatographic paper using a laser cutter with a precision of ±0.1 mm and a cutting speed of 200 mm/s. In less than 5 s, the hydrophobic barriers were fabricated on the paper, leading to the creation of a final device of 30 mm in length. The paper-based device was composed of a circular central zone (Ø = 7 mm) and three detection zones (Ø = 7 mm) for glucose, lactate, and cholesterol, respectively, connected through three microfluidic channels (14 mm in length). Three pre-treatment zones (Ø = 3.5 mm) were interposed between the sample and the test zones.

### 2.3. Multibranched Gold Nanoparticles Preparation and Colorimetric Assay

Colloidal 60 nm multibranched gold nanoparticles (MGNPs) were synthesized according to a procedure previously optimized in our group [18]. A blue colloidal suspension was characterized by DLS, UV-vis, and TEM. To achieve optical detection via naked eye, a concentrated of MGNP was suspended in MilliQ water (OD 12 @648 nm) to provide a vivid and well distinguishable color both before and after the reaction. This detection strategy relies on particle reshaping mediated by NaI and H_2_O_2_ (produced by analyte-oxidase reaction). The selective erosion of nanoparticle tips allows for a blue LSPR shift and a visible color change from blue to pink while avoiding any OD loss.

### 2.4. Assay Procedure and Data Collection

The paper-based multiplexed device was functionalized for the simultaneous colorimetric detection of glucose, lactate, and cholesterol. In the pre-treatment zone, the surface of the paper was first adsorbed with 1.5 µL of NaI. The halogen, which was solved in an aqueous solution of sodium phosphate buffer pH = 6, 100 mM, was prepared in a concentration dependent on the specific detection assays: 250 µM for glucose test, 150 µM for lactate, and 100 µM for cholesterol. Each condition was selected so as to respond in a relatively short time (10 min) in the physiological concentrations range of the three analytes, leading to a visible outcome of pathological conditions. The detection zones were treated with 0.5 µL of MGNPs and a layer (1.5 µL) of the oxidase enzyme was placed on the MGNP surface. Each of the three sensing areas was functionalized with the specific oxidase enzyme for the detection of the three analytes: glucose oxidase (145 U, pH = 7, 100 mM), lactate oxidase (0.25 U, pH = 7, 50 mM) and cholesterol oxidase (2.5 U, pH = 7, 50 mM). After the device was dried under vacuum for 15 min, saliva (40 µL) containing different concentrations of the three analytes was added to the sample zone until it reached the testing zones. After the test (10 min), the colorimetric signals were recorded using a smartphone, standardizing the image acquisition, whereby illumination was provided by an external lamp, devices were placed on a white and matt photography backdrop, and the smartphone was located on a support ensuring its equal distance from the light source and the multiplexed device. Ultimately, all the pictures were processed through ImageJ software, recording red coordinate of RGB color space, which was the most effective in interpreting the color change of the assay. All the assays were performed on 10 real salivary samples and the correspondent red values represent the average of 3 independent measurements performed for each sample.

### 2.5. Sample Preparation

The samples used in this work included spiked saliva samples provided by healthy subjects. Here, the use of saliva was approved by Ethical Committee of Regione Liguria (405/2020-DB id 10787). Eating, drinking and oral hygiene procedures were prohibited for at least 1 h before the collection as unstimulated saliva was required for the reliability of the test [18,33]. We employed saliva samples from 10 different donors; for safety, we manipulated the samples under the chemical hood, filtering them with a 0.2 µm methyl cellulose syringe filter to remove bacteria. Pathological conditions were simulated by adding to the collected saliva aqueous solutions of glucose (14 and 28 µM) and lactate (2 and 4 mM). The cholesterol solution (5.2 µM) was obtained by dissolving cholesterol powder in 10% Triton X-100, and they were diluted in a 50 mM sodium phosphate buffer. Non-supplemented saliva, representing physiological normal conditions, was used as a control.

## 3. Results and Discussion

### 3.1. Device Configuration and Working Mechanism

**Figure 1:**
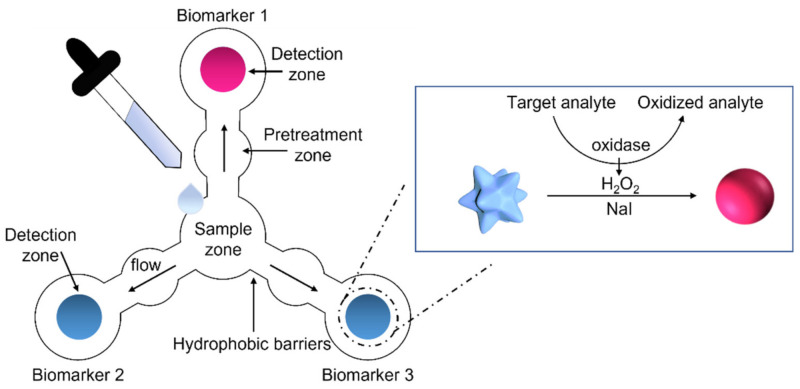
Schematic illustration of the monolithic paper-based device for the simultaneous detection of three salivary biomarkers. The device was realized using a CO_2_ laser cutter. A drop of saliva is deposited in the central area (sample zone). Flowing in a microfluidic pattern, saliva passes through the pretreatment zones, where it is mixed with the deposited halogen (NaI), after which it reaches the detection zones, previously spotted with the blue colloidal suspension of MGNPs, functionalized with a layer of oxidase enzymes, one for each sensing area. The insert shows the colorimetric detection strategy that relies on the target-induced reshaping of plasmonic MGNPs, mediated by H_2_O_2_ (byproduct of the biomarker oxidation by the specific oxidase enzyme), in presence of NaI. This reaction leads to a visible color change from blue to pink for non-physiological levels of the target analyte.

The proposed device was designed to allow for a multiplexed diagnosis of three model biomarkers in saliva (its schematic description is reported in Figure 1). Chromatography paper was used as the substrate, and a graphic software was employed to project a fully integrated microfluidic device, using a single drop of saliva. The final design of the device consists of a small central area (sample zone) where the saliva is casted, three sensing areas (detection zones) spotted with gold nanoparticles (GNPs) and a layer of a specific enzyme, three independent channels, and three regions placed in the middle of each branch (pretreatment zones) functionalized with a halogen species (i.e., NaI). Each sensing area is responsible for the detection of a specific salivary biomarker, namely, cholesterol, glucose, or lactate. Upon the deposition of a few microliters of saliva in the sample zone, the biological fluid is transported through the microfluidic channels, and is homogeneously distributed in each of the three branches. After passing through the pretreatment zones, saliva is mixed with NaI, which is a crucial reagent for the final detection reaction. In the last step, saliva reaches the detection zones, where plasmonic GNPs are deposited. In particular, the sensing scheme relies on 60 nm multibranched GNPs (MGNPs) that exhibit an intense blue color, due to their peculiar shape of several small tips on the particle surface [34]. For the detection of the three proposed analytes, a layer of the correspondent oxidase enzyme (cholesterol oxidase (ChOx), glucose oxidase (GOx), and lactate oxidase (LOx)) was placed on the MGNP surface, one for each sensing area. As illustrated in the inset of Figure 1, the colorimetric reaction involves the oxidation of the biomarker by the specific enzyme, which generates hydrogen peroxide (H_2_O_2_) as a byproduct. H_2_O_2_ is converted into its free radical form, which rapidly etches the MGNPs, leading to their morphological change into spherical NPs, thus promoting a color shift from blue to pink [18]. The process does not involve optical density loss and occurs within few minutes due to the presence of the halogen, which boosts the oxidation process, promoting a rapid color change. The reshaping process was finely tuned in order to respond in the physiological range of the target’s concentration and to provide a predictive color change in pathological conditions.

The analytical device developed in this work was realized using a paper sheet via a CO_2_ laser cutter instrument. This approach allows for the production of the designed pattern with hydrophobic boundaries in a single step and with high resolution. CO_2_ laser ablation enables fine feature definition, avoiding multistep fabrication protocols, chemical treatments, or organic solvent use. Furthermore, this method has proven to be less expensive than other bench-top approaches, while allowing for the rapid parallelization of the procedure and, hence, a certain scalability to medium-level production. In our study, we investigated several experimental parameters for device fabrication, especially in terms of laser power and beam speed, since high power can lead to burned edges and a decreased width of the cellulose channels, whereas low levels of power cannot guarantee the correct formation of the hydrophobic boundaries. After some optimizations, a power of 12 W and a speed of 50 mm/s were selected. Interestingly, from a single paper sheet, we could produce around 12 devices in less than 1 min, maintaining high cutting precision and a lateral resolution of 1 µm (see Figure S1).

After the realization of the monolithic multiplexed device, the paper was spotted in the pretreatment zones with a solution of NaI, with a specific concentration in each branch, depending on the proposed biomarker detection assay (see also below), while equal amounts of MGNPs were placed in the detection zones, to achieve the same color intensity of the spots. Plasmonic particles were then covered by a layer of the specific oxidase enzyme at varied concentrations, as a function of the required sensitivity. As mentioned above, the colorimetric reaction relies on a particle reshaping mechanism, resulting in a clear plasmonic shift (TEM micrographs of MGNPs before and after the assay performed on saliva spiked with a non-physiological amount of glucose are provided in Figure S2). This strategy allows for the high detection sensitivity that is necessary for rapid (10 min) analyte detection for salivary concentrations.

In this work, we aim to simultaneously detect glucose, cholesterol, and lactate. They are model targets of clinical importance, which all play important roles in the metabolic processes [35,36,37]. High concentration of glucose leads to hyperglycemia and diabetes. Accumulation of lactate can cause muscle ache and fatigue and its continuous monitoring could be predictive of athletic performance [38]. Abnormal levels of cholesterol are related to hypercholesterolemia. A simultaneous determination of these clinical biomarkers can thus provide important information for health monitoring and early diagnosis. Additionally, the use of saliva as a biological source provides many considerable advantages: first, a large variety of well-known disease-related biomarkers, including glucose, cholesterol, and lactate, are normally present in saliva; second, it offers a non-invasive, simple, and rapid (auto)sampling process, opening the route to frequent POC screenings for early diagnosis and personalized treatments, and a reduced risk of infections.

### 3.2. Assay Optimization

Preliminary optimizations of the device configuration, alongside an optimization of the concentrations and volumes of reagents and nanoparticles were performed to obtain a suitable POCT with a clear colorimetric readout for each assay in a short time period (10 min). After designing the hydrophobic channels on the cellulose paper, varying volumes (20–50 µL) of a saliva sample, mixed with a red dye, were deposited on the central zone to investigate the multiplexing distribution ability of the device. We found that a 40 µL solution was sufficient to cover the detection zone, with no overflow. Importantly, the saliva specimen homogeneously diffused from the center to the detection zones through the capillary forces in less than 10 sec (Figure S3), with a slower rate in correspondence of the wider pretreatment zones for the Bernoulli’s principle [39,40]. To promote a uniform release and diffusion of the sample in each branch of the device, saliva was deposited on a round piece (Ø = 7 mm, cut with a puncher) of Fusion 5, placed in the deposition zone. This type of single layer matrix, commonly used in lateral flow devices, enables the conjugate release, fast flow, and sample wicking without a loss of sensitivity.

Different pH levels, buffers, and enzyme concentrations were also investigated to optimize the detection performance of each analyte (see also Methods for details and Figures S4–S6). Furthermore, the concentration of the halogen was refined as follows: for the glucose test, a concentration of 250 µM was selected, as it allows for reshaping in a short time period, while avoiding false positives as a result of a-specific etching. Similarly, 150 and 100 µM were established for lactate and cholesterol assays, respectively. Each condition was chosen to provide a clear optical readout in ca. 10 min, with a color change in case of above-physiological concentrations of the three biomarkers. To achieve this aim, a salivary threshold was assessed for each assay after validating the corresponding blood value from the literature. To mimic the non-physiologically relevant conditions, saliva was spiked with 5.2 µM of cholesterol (>200 mg/dL in blood), 14 µM of glucose (>130 mg/dL in blood) and 2 mM of lactate (>4 mM in blood) [35,36,41]. Furthermore, for the glucose and lactate analyses, an additional non-physiological level was included in the higher concentration range. In case of hyperglycemia, it could be of clinical importance to detect whether the level of glucose slightly exceeds the pathological concentration, or if it is significantly higher. Hence, 28 µM could be considered as another interesting range, corresponding to a blood value > 200 mg/dL. Likewise, for an athlete, it could be interesting to evaluate the training progress, the intensity of exercise, or to assess the cardiovascular and pulmonary health, thus we determined a further above-threshold value of 4 mM (typically referred to as “maximal lactate steady state”) [42].

Concerning the optical detection via naked-eye or smartphone, the MGNP concentration was tuned to provide a vivid and distinctive color both before and after the reaction. The particle suspension, 0.5 µL in volume, was deposited via direct drop casting on the paper device. MGNPs were placed in the middle of the detection zone to ensure that the saliva samples reach the test zone and homogeneously mixed with all the reagents, providing a clear and uniform colored spot.

### 3.3. Analytical Performance

**Figure 2:**
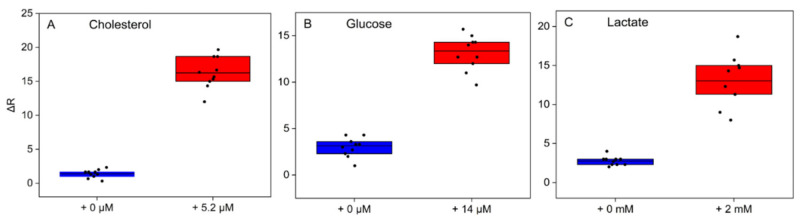
ΔR values of the detection zones collected at the end of the assay (10 min) performed on native saliva (blue bars) and saliva spiked with (**A**) 5.2 µM cholesterol, (**B**) 14 µM glucose and (**C**) 2 mM lactate (red bars). Graphs provide the average result of 10 assays performed on saliva collected from 10 different donors.

Under the optimized experimental conditions, we first tested the colorimetric biosensor for the individual determination of glucose, cholesterol, and lactate. For each assay, saliva samples from 10 different donors, native and spiked with a standard solution of the proposed target, were added in the deposition zone of the devices. Following image acquisition at the starting point and at the end of the assay (10 min) using a standard smartphone, the ΔR values (of RGB coordinates) of the three detection zones were collected and analyzed with ImageJ software. The ΔRs of the native samples are plotted against the spiked samples in Figure 2. As the concentration of the biomarker in saliva surpasses the established pathological threshold, a significant color variation (from blue to pink) was observed by both the naked eye and ΔR values. While some heterogeneity among the different samples as well as some variability in the reaction rate were recorded when using saliva from different donors, the ON/OFF outcome was not affected for all the biomarkers tested. As can be seen in Figure 2, for all the three target assays, the native saliva presents negligible red coordinate variations, with R values always confined in an ΔR range between 0 and 5. From this evidence, we can conclude that false positives are excluded. On the contrary, salivary samples spiked with pathological concentrations of the three analytes are, on average, grouped around the same ΔR value, showing a remarkable color distinction compared with the controls in terms of red coordinates, which reflects a visible color shift by the naked eye.

**Figure 3:**
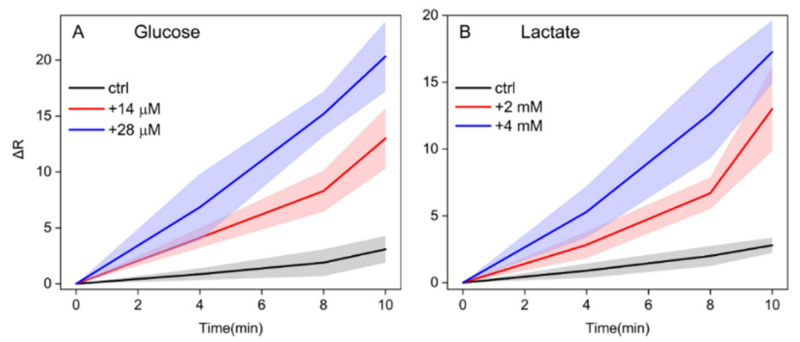
ΔR values evolution over time of the detection zones after the assays on native saliva (black line), saliva spiked with the lowest (red line) and the highest (blue line) established non-physiological concentrations of (**A**) glucose (respectively, +14 and +28 µM) and (**B**) lactate (respectively, +2 and +4 mM). The colored bands represent the standard deviation of 10 measurements performed on different salivary samples.

Moreover, we investigated the semiquantitative response ability of the assay, since understanding if the levels of glucose and lactate significantly exceed the basal level could be of great interest. Ten samples, spiked with 14 and 28 µM of glucose, were therefore tested on the proposed device. The evolution of the colorimetric response over time is plotted in Figure 3A. Likewise, 10 samples, with increased 2 and 4 mM lactate solutions, were tested against the native saliva (Figure 3B). RGB coordinates were collected every 2 min by a smartphone. For the highest concentration of the two analytes, the red coordinate of the detection zones immediately raised, leading to a visually appreciable, positive result after only 4–5 min. On the other hand, for the lowest non-physiological concentration, the spot began changing its color only after 7–8 min, demonstrating that a time delay is required for processing a reduced amount of the target. Instead, non-spiked control saliva consistently showed an ΔR < 5 after 10 min, proving the specificity of the assay and the absence of false positives.

### 3.4. Selective Multiplexing Detection of the Three Biomarkers

**Figure 4:**
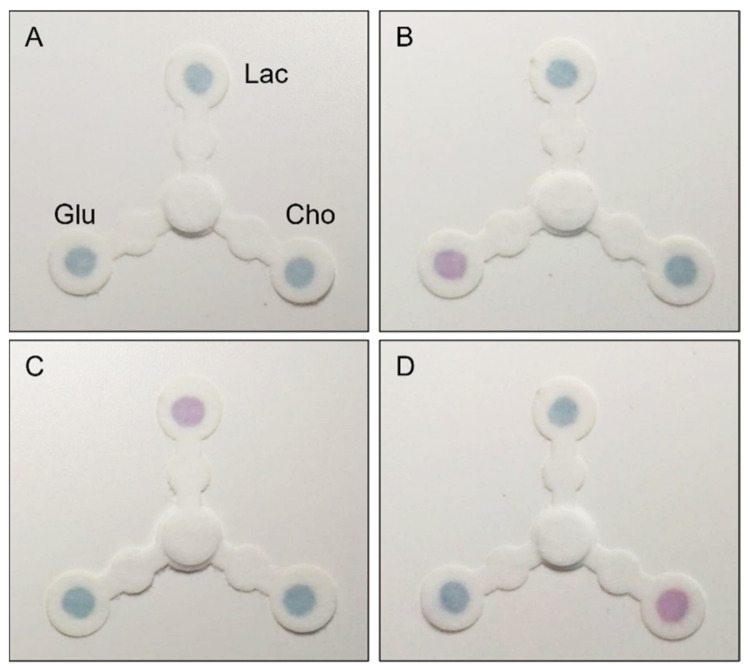
Representative photographs of the multiplexed devices after 10 min of reaction, treated with (**A**) native saliva, (**B**) saliva spiked with glucose (Glu) (14 µM), (**C**) saliva spiked with lactate (Lac) (2 mM) and (**D**) saliva spiked with cholesterol (Cho) (5.2 µM).

Under the optimal assay conditions, the analytical performance of the multiplexed device for the selective colorimetric detection of cholesterol, glucose, and lactate was investigated. The deposition zones of four different devices were treated with (A) native saliva, (B) saliva spiked with glucose, (C) with lactate, and (D) with cholesterol (Figure 4). After 10 min, a distinct pink color appeared in the specific detection zones, in which non-physiological concentrations of the specific analyte were present. On the contrary, the remaining two sensing areas were not affected by color change and remained blue, proving the selectivity of the assay and the non-interference of the analytes between them.

**Figure 5:**
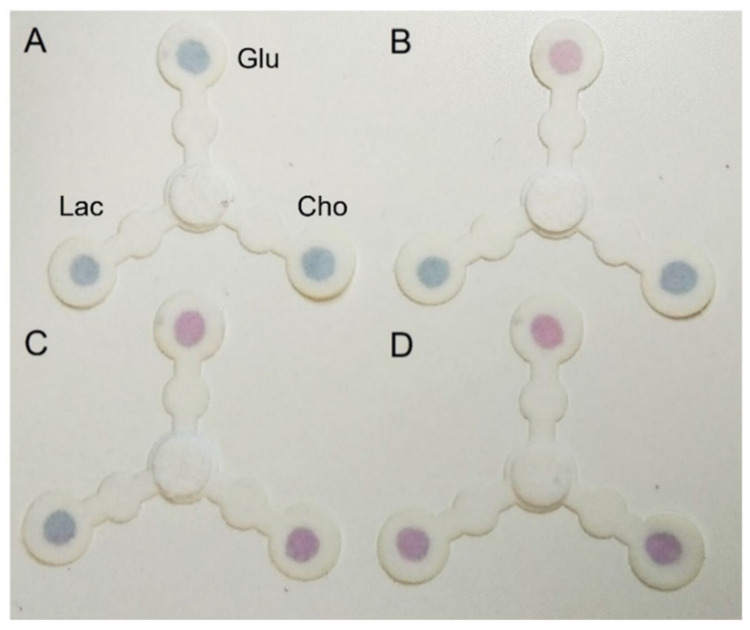
Photographs of four devices at the end of the reaction (10 min) treated respectively with (**A**) native saliva, (**B**) saliva spiked with one target biomarker (14 µM glucose (Glu)), (**C**) saliva spiked with two targets (14 µM glucose + 5.2 µM cholesterol (Cho)), and (**D**) saliva spiked with all the targets (14 µM glucose + 5.2 µM cholesterol + 2 mM lactate (Lac)).

Next, the simultaneous detection of glucose, cholesterol, and lactate in saliva was analyzed. Four different samples were prepared, namely, saliva, saliva spiked with glucose, saliva with glucose and cholesterol, saliva with all the target analytes. Figure 5 presents the results after the reaction: the detection zones were sensitive to the specific targets. Remarkably, when all the biomarkers were simultaneously present above the physiological levels, the device exhibited a distinct pink color in all the three sensing areas. Additionally, false positives were excluded when physiological conditions (no spiked saliva) were tested. These results prove that this multiplex paper-based system is applicable for the simultaneous monitoring of cholesterol, glucose, and lactate in real samples. Despite the accumulation of these targets may lead to the occurrence of relevant diseases, we must recall that they only represent model biomarkers, while several other pathologies, directly involving different oxidase enzymes, could be monitored with the proposed sensing platform.

### 3.5. Interference Assessment

**Figure 6:**
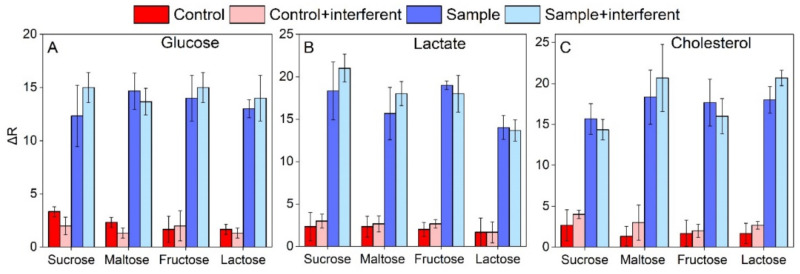
ΔR values collected at the end of the assay (10 min) performed on native saliva (red histograms), saliva with interferents (pink histograms), saliva spiked with (**A**) 14 µM glucose, (**B**) 2 mM lactate, and (**C**) 5.2 µM cholesterol (dark blue histograms), and saliva spiked with a mix of the analytes and the interferents (light blue histograms). Error bars represents the uncertainty from three independent measurements.

The interferences of compounds possibly present in saliva were evaluated for the three target analytes. Aqueous solutions of sucrose, mannose, lactose, and fructose were added to the sample one by one. Four paper-based devices were functionalized with glucose oxidase and treated with native saliva, saliva with interferent, saliva with glucose, and saliva with glucose and the interferent, respectively. The same procedure was applied for the cholesterol and lactate. ΔR values of saliva, spiked with the interferent species, were plotted compared to those of non-spiked samples. As shown in Figure 6, for all the compounds evaluated and for all the analytes detected, ΔRs did not show remarkable variations, indicating that the interference of the tested species was minimal.

### 3.6. Prototype Kit

**Figure 7:**
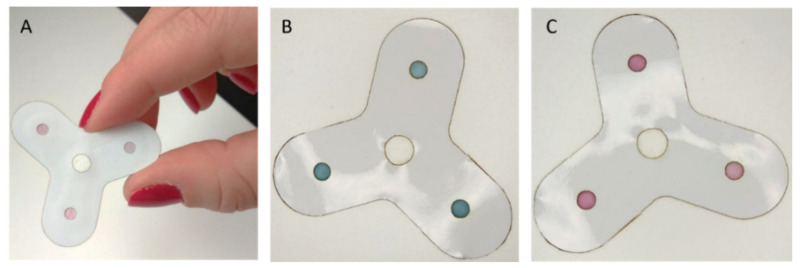
(**A**) Photograph of the final device embedded in the PVC mask with, representative of its real size. (**B**,**C**) Images of the proposed prototype with the specific mask after the assay on saliva spiked with the three analytes at the beginning of the test (**B**) and after 10 min of reaction (**C**).

Additionally, after validating the multiple detection ability of the device, we demonstrated the feasibility to realize a prototype kit for POCT testing. A sheet of polyvinyl chloride (PVC) adhesive film, designed with holes in correspondence of the deposition and detection zones, was employed as a protective film on the paper surface. The shape and pattern of this inexpensive layer were also fabricated using the laser cutter. The paper layer was placed between the PVC film and an adhesive sealing film, achieving a sandwich-like structure. The mask, acting as a protective film on the surface, provides versatility and handling to the paper-based device (Figure 7). We further confirmed the multiplex detection ability of this prototype, performing the assay on saliva spiked with the three analytes. Figure 7B shows the device at the starting point (immediately after the deposition of the saliva sample) and after the duration of the test (Figure 7C).

This result provides ways forward for the realization of a robust and user-friendly POCT for non-skilled operators.

## 4. Conclusions

In this work, a monolithic and fully integrated paper-based device for the multiplexed detection of glucose, cholesterol, and lactate in saliva was developed. The pattern of the developed device was realized using commercially available graphic software and transferred on chromatography filter paper using a CO_2_ laser cutter. In a single step, and in less than 5 s, the fluidic pattern was produced on the paper, and the analytical performance of the device was tested on different salivary samples. By exploiting the reshaping of plasmonic gold nanoparticles, we could achieve, in 10 min, a visual qualitative readout, appreciable by both the naked-eye and a smartphone camera, achieving the high sensitivity required for detecting the low concentration of salivary biomarkers. The analytical device was proven to provide excellent selectivity for single or simultaneous detection of the proposed biomarkers, and we were able to exclude the interference from species commonly present in saliva. We further created a prototype kit for POCT testing, developing a low cost and robust PVC mask suitable for home-testing of health status and for monitoring athletic performances. This platform could be adapted for the monitoring of several other biomarkers and pathologies or could be morphologically implemented to increase the number of targets simultaneously detected.

## Supplementary Materials

The following are available online at https://www.mdpi.com/article/10.3390/bios11110443/s1, Figure S1: paper devices fabricated by CO_2_ laser cutting, Figure S2: TEM images of the reshaping process, Figure S3: Evolution of salivary flow, Figures S4–S6: optimization experiments of the microfluidic device.
