# Supplementary material for: Paper-Based Multiplexed Colorimetric Device for the Simultaneous Detection of Salivary Biomarkers
Source: Biosensors (Basel). 2021 Nov 10;11(11):443. doi: 10.3390/bios11110443 (PMC8615519; doi:10.3390/bios11110443)
Supplement: Supplementary file 1 [file biosensors-11-00443-s001.zip › biosensors-1440704-supplementary.pdf]

Paper-based multiplexed colorimetric device for the simultaneous detection of salivary biomarkers

Tania Pomili[1,2], Paolo Donati[1] and Pier Paolo Pompa[1*]

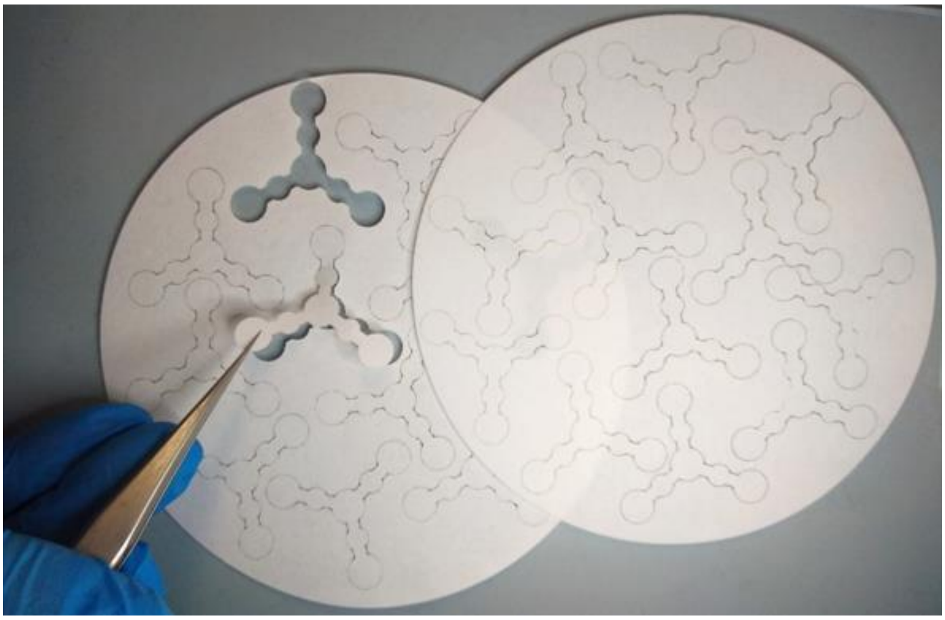

Figure S1. Representative photograph of two paper sheets after CO_2 laser cutting treatment for the realization of the designed microfluidic paper-based multiplexed devices. A single three-branch device is typically fabricated in ca. 5 sec.

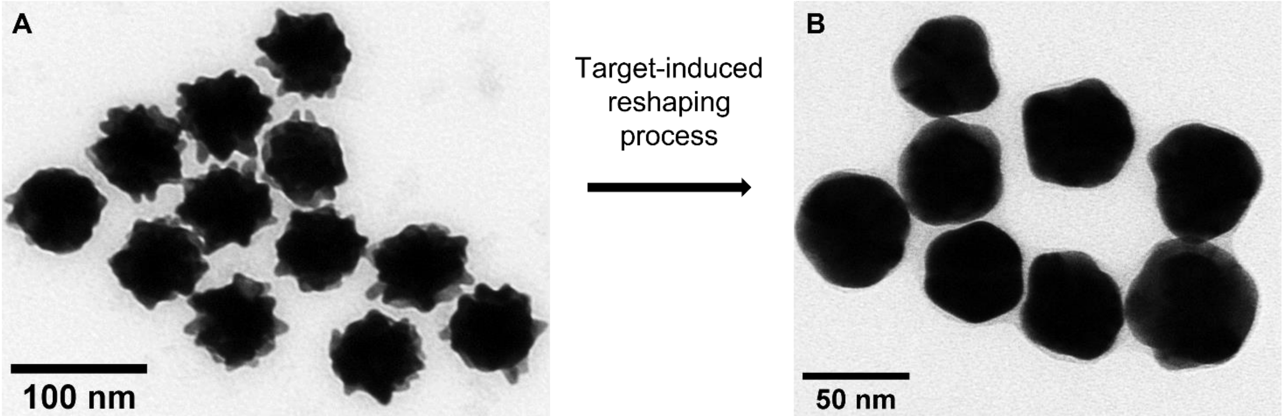

Figure S2. TEM micrographs of the MGNPs before (A) and after (B) the assay with saliva spiked with glucose, simulating non-physiological condition (glucose was chosen as model biomarker for displaying the reshaping process).

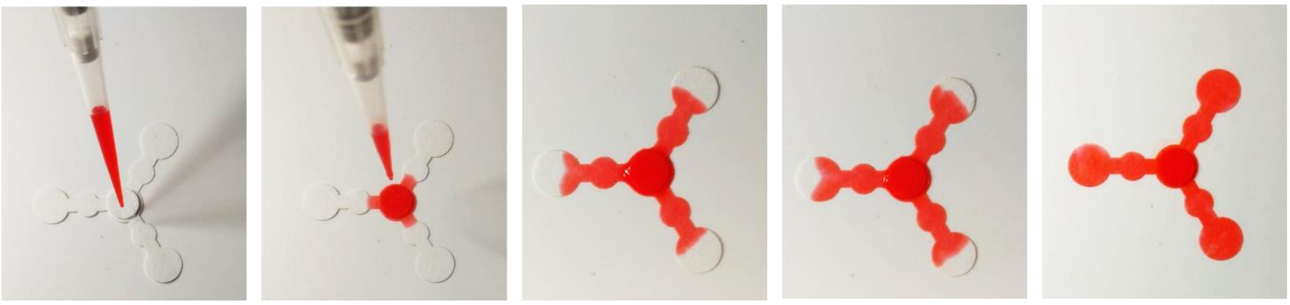

Figure S3. Over time evolution of the flow of saliva, mixed with a red dye, on the designed paper-based device. 40 µL of saliva were drop casted onto the sample zone, flowing through the microfluidic patterns confined inside the hydrophobic boundaries. As it is clearly shown, an isotropic evolution of the salivary flow occurred in all the three branches of the device. In less than 10 seconds, the saliva reached the detection zones and resulted uniformly distributed on all the detection regions.

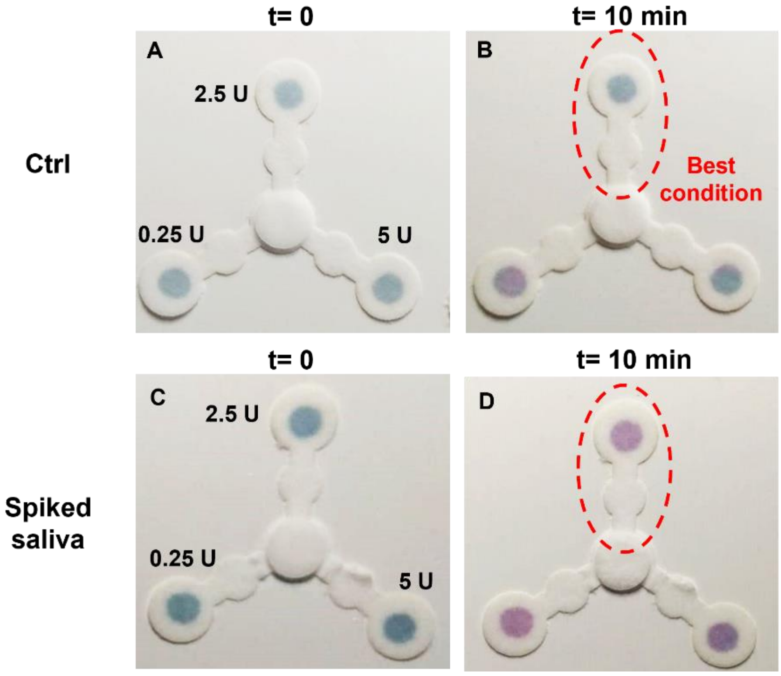

Figure S4. Optimization of the enzyme concentration in the device. Devices were functionalized with 0.25, 2.5, and 5 units of cholesterol oxidase. Native saliva (A and B) and saliva spiked with 5.2 µM cholesterol (C and D) were tested, and photographs were collected at the starting point (t=0) and at the end of the reaction (t=10 minutes). When the lower enzyme concentration (0.25 U) was used, nanoparticles were not sufficiently covered by the protein layer, resulting in aspecific etching and leading to color change even with no spiked saliva (B panel). Among the remaining two concentrations, 2.5 U gave a better color when spiked saliva was tested (D) and, hence, it was chosen as the best condition. The same experiments were performed for glucose (73, 145, and 290 U tested) and lactate (0.12, 0.25 and 1 U tested), giving similar behavior.

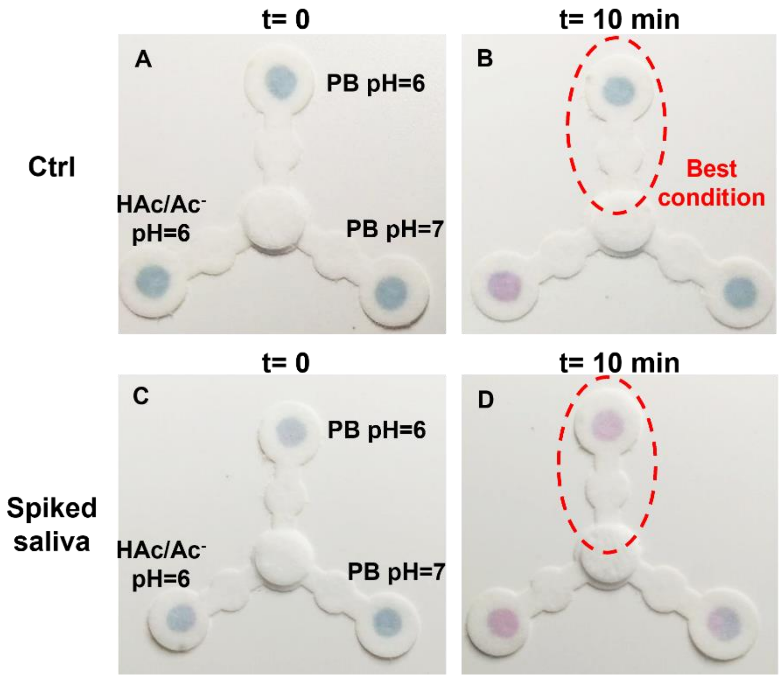

Figure S5. Optimization of the buffer used for NaI solution. Devices were functionalized with lactate oxidase and three solutions of NaI solved in acetate buffer pH=6, 100 mM, sodium phosphate buffer pH=6, 100 mM, and sodium phosphate buffer pH=7, 100 mM. Native saliva (A) and (B) and saliva spiked with 2 mM lactate (C) and (D) were tested, and photographs were collected at the starting point (t=0) and at the end of the reaction (t=10 minutes). Acetate buffer promoted a quick LSPR shift also in the control samples, due to aspecific etching (B). Sodium phosphate buffer was tested at two pH conditions and, as it is clearly illustrated in (D), pH=6 gave a faster reshaping process and an intense color change, so it was chosen as the best condition. The same experiments were performed for cholesterol and glucose, confirming this evidence.

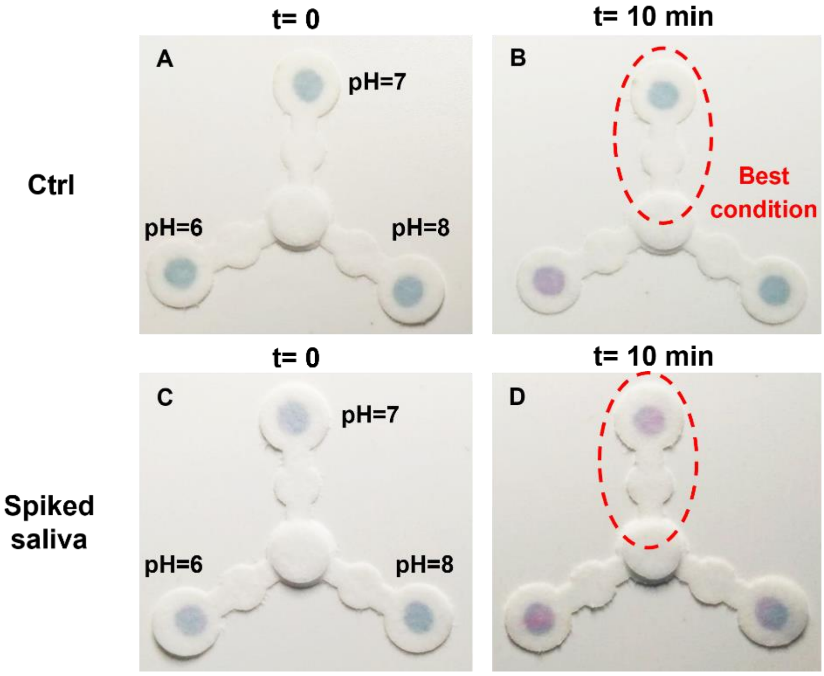

Figure S6. Optimization of the pH used for enzyme solution. Devices were functionalized with glucose oxidase solved in sodium phosphate buffer, 100 mM, pH=6, pH=7, and pH=8. Native saliva (A) and (B) and saliva spiked with 14 µM glucose (C) and (D) were tested, and photographs were collected at the starting point (t=0) and at the end of the reaction (t=10 minutes). The most acidic conditions (pH=6) boosted the reshaping process, leading to false positive results (B). On the contrary, basic solutions slowed down the reshaping, giving unclear color changes after 10 minutes (D). Based

on such evidences, pH=6 was chosen as the best condition. The same experiments were performed for lactate and cholesterol, confirming the results.
